# Population genomics of pneumococcal carriage in South Africa following the introduction of the 13-valent pneumococcal conjugate vaccine (PCV13) immunization

**DOI:** 10.1099/mgen.0.000831

**Published:** 2022-06-23

**Authors:** Nida Javaid, Courtney Olwagen, Susan Nzenze, Paulina Hawkins, Rebecca Gladstone, Lesley McGee, Robert F. Breiman, Stephen D. Bentley, Shabir A. Madhi, Stephanie Lo

**Affiliations:** ^1^​ Department of Biology, Syed Babar Ali School of Science and Engineering, Lahore University of Management Sciences, Lahore, Pakistan; ^2^​ Parasites and Microbes, Wellcome Sanger Institute, Hinxton, UK; ^3^​ Department of Science and Technology/National Research Foundation: Vaccine Preventable Diseases, University of the Witwatersrand, Johannesburg, South Africa; ^4^​ Medical Research Council: Respiratory and Meningeal Pathogens Research Unit, School of Pathology, Faculty of Health Sciences, University of the Witwatersrand, Johannesburg, South Africa; ^5^​ Centers for Disease Control and Prevention, Atlanta, GA, USA; ^6^​ Emory Global Health Institute, Atlanta, USA; ^7^​ Department of Pathology, University of Cambridge, Cambridge, UK

**Keywords:** antimicrobial resistance, pneumococci, pneumococcal carriage, pneumococcal conjugate vaccine, pneumococcal genomics

## Abstract

*
Streptococcus pneumoniae* is a major human pathogen responsible for over 317000 deaths in children <5 years of age with the burden of the disease being highest in low- and middle-income countries including South Africa. Following the introduction of the 7-valent and 13-valent pneumococcal conjugate vaccine (PCV) in South Africa in 2009 and 2011, respectively, a decrease in both invasive pneumococcal infections and asymptomatic carriage of vaccine-type pneumococci were reported. In this study, we described the changing epidemiology of the pneumococcal carriage population in South Africa, by sequencing the genomes of 1825 isolates collected between 2009 and 2013. Using these genomic data, we reported the changes in serotypes, Global Pneumococcal Sequence Clusters (GPSCs), and antibiotic resistance before and after the introduction of PCV13. The pneumococcal carriage population in South Africa has a high level of diversity, comprising of 126 GPSCs and 49 serotypes. Of the ten most prevalent GPSCs detected, six were predominantly found in Africa (GPSC22, GPSC21, GPSC17, GPSC33, GPSC34 and GPSC52). We found a significant decrease in PCV7 serotypes (19F, 6B, 23F and 14) and an increase in non-vaccine serotypes (NVT) (16F, 34, 35B and 11A) among children <2 years of age. The increase in NVTs was driven by pneumococcal lineages GPSC33, GPSC34, GPSC5 and GPSC22. Overall, a decrease in antibiotic resistance for 11 antimicrobials was detected in the PCV13 era. Further, we reported a higher resistance prevalence among vaccine types (VTs), as compared to NVTs; however, an increase in penicillin resistance among NVT was observed between the PCV7 and PCV13 eras. The carriage isolates from South Africa predominantly belonged to pneumococcal lineages, which are endemic to Africa. While the introduction of PCV resulted in an overall reduction of resistance in pneumococcal carriage isolates, an increase in penicillin resistance among NVTs was detected in children aged between 3 and 5 years, driven by the expansion of penicillin-resistant clones associated with NVTs in the PCV13 era.

## Data Summary

Whole-genome sequences were deposited at European Nucleotide Archive (ENA) and the accession numbers are available in the metadata (Table S1, available in the online version of this article). The Phylogenetic snapshot of carriage isolates from South Africa is available at https://microreact.org/project/GPS_South_Africa_carriage. The authors confirm that all supporting data, code, and protocols have been provided within the article or through supplementary data files.

**Impact Statement**

This is the first study to report the population epidemiology of pneumococcal carriage isolates in South Africa at the genomic level and describes the changes in pneumococcal serotypes, lineages and antimicrobial resistance before and after the introduction of PCV7/13, in a setting with a high HIV disease burden. The study identified pneumococcal lineages that were associated with increased colonization by non-vaccine serotypes. Further, an increase in penicillin resistance in NVT isolates from children aged 3 to 5 years was detected, which may potentially become a public health challenge.

## Introduction

*Streptococcus pneumoniae *, also known as the pneumococcus, is a major human pathogen responsible for causing a range of diseases from mild infections (e.g. otitis media and bronchitis) to life-threatening diseases (e.g. pneumonia, meningitis and septicaemia) [1]. In 2015, it was estimated to be responsible for 9 million cases of disease and over 317000 deaths in children under 5 years of age, with the main disease burden in low- and middle-income countries (LMIC) [2]. Nasopharyngeal carriage of pneumococci is a prerequisite of pneumococcal infections and is an important indicator of pneumococcal population dynamics [1, 3].

Current vaccines against *S. pneumoniae* target the more prevalent, invasive and antibiotic-resistant serotypes, and when included in the childhood immunization programmes have shown great success in reducing the burden of under-5 childhood morbidity, including reductions in invasive pneumococcal disease (IPD) and all-cause pneumonia hospitalization [4]. While PCV immunization effectively reduces the carriage of vaccine serotypes (VT), there is little effect on the overall pneumococcal carriage rates, suggesting the expansion of non-vaccine serotypes (NVT) to fill the vacant niche, a phenomenon known as serotype replacement [5]. In South Africa, in the pre-vaccine era, carriage rates by PCV7 serotypes in children under the age of 2 ranged between 23–39 % for children living with HIV and between 25–38 % in HIV-uninfected children [6]. South Africa introduced PCV7 and PCV13 in the routine infant immunization programme in 2009 and 2011, respectively [7]. Following the introduction of PCVs in South Africa, significant decreases in IPD [8] and pneumococcal colonization [9, 10] were observed. The latter was mainly due to a significant decrease in VT colonization, with a relatively modest increase in NVTs (e.g. 11A, 15A, 16F, 21, 34 and 35B) [9, 10]. This decreasing trend was also observed amongst children ≤12 years of age living with HIV [11].

To better understand the impact of PCV on the pneumococcal carriage population, this study aimed to investigate the change in circulating pneumococcal strains, and related serotypes and antibiotic resistance before and after PCV7/13 introduction, by sequencing the genomes of a subset of carriage isolates collected in previous cross-sectional surveys conducted in South Africa [9–11].

## Methodology

### Study design

*S. pneumoniae* isolates were previously collected in cross-sectional carriage surveys at two sites: Soweto (an urban township of Johannesburg) and Agincourt (a town in rural North-East), South Africa [9–11]. In Agincourt, households with children ≤2 years of age were randomly sampled, and nasopharyngeal swabs were collected from the index child, together with at least one individual older than 12 years in the same household in the year of 2009, 2011 and 2013 [9, 10]. In Soweto, the survey enrolled mother–child dyads with or without underlying HIV in 2010–2011 and then in 2012–2013 [11]. These two regions are of high HIV prevalence in adults, including 37.4 % in Agincourt (2011) and 30 % among women in Soweto [10, 11]. A decrease in HIV prevalence in children has been reported due to decreased mother-to-child HIV transmission following antiretroviral therapy [9, 11]. Metadata including age, gender, region, HIV status and date of sample collection was collected. As part of the Global Pneumococcal Sequencing project (GPS, www.pneumogen.net), 1825 archived pneumococcal isolates collected in these surveys were randomly selected for whole-genome sequencing (Table 1).

**Table 1.** Demographics of the pneumococcal dataset in this study

Characteristics	Percentage (*n*/*N*)		
	Agincourt	Soweto	Total
***n***	1097	728	1825
**Gender**			
Female	50.3 % (552/1097)	58.8 % (426/725)	53.7 % (978/1822)
Male	49.7 % (545/1097)	41.2 % (299/725)	46.3 % (844/1822)
**Age groups (year**)			
≤2	57.8 % (634/1097)	49.6 % (361/728)	54.5 % (995/1825)
3–5	18.2 % (200/1097)	26.1 % (190/728)	21.37 % (390/1825)
>5	24 % (263/1097)	24.3 % (177/728)	24.1 % (440/1825)
**HIV status**			
HIV-infected	93.2 % (357/383)	50.1 % (364/727)	64.9 % (721/1110)
HIV-uninfected	6.8 % (26/383)	49.9 % (363/727)	35.1 % (389/1110)
**Year**			
2009	34.1 % (374/1097)	–	20.5 % (374/1825)
2010	–	49.5 % (360/728)	19.7 % (360/1825)
2011	32.6 % (358/1097)	–	19.6 % (358/1825)
2012	–	47.5 % (346/728)	19.0 % (346/1825)
2013	33.27 % (365/1097)	3.0 % (22/728)	21.2 % (387/1825)
**Vaccine era**			
PCV7	49.1 % (539/1097)	49.5 % (360/728)	49.2 % (899/1825)
PCV13	50.9 % (558/1097)	50.5 % (368/728)	50.8 % (926/1825)

Samples were grouped into two vaccine periods based on the date of sample collection: (1) PCV7 era: included samples collected between June 2009 and July 2011 (after the introduction of PCV7 but before PCV13), (2) PCV13 era: included samples collected between August 2011 and November 2013 (after the introduction of PCV13).

### Genomic sequencing and analysis

Pneumococcal genomes were sequenced on an Illumina HiSeq platform, which produced paired-end sequence reads of 151 bps in length, and quality control on sequence reads, assemblies and annotation was performed as previously described [12, 13]. Serotypes and sequence types (STs) were inferred from the genome data using SeroBA [14] and MLSTcheck [15]. A clonal complex (CC) was defined as a group of STs with a single locus variance within the GPS dataset (*n*=13 454) using E-burst [12]. Antibiotic resistance against 17 antibiotics, including penicillin, amoxicillin, meropenem, cefotaxime, ceftriaxone, cefuroxime, erythromycin, clindamycin, synercid, linezolid, cotrimoxazole, tetracycline, doxycycline, levofloxacin, chloramphenicol rifampin and vancomycin, was predicted from the genomic data using the CDC’s Streptococcus Laboratory pneumococcal typing pipeline [16–18]. Minimum inhibitory concentrations were interpreted using Clinical and Laboratory Standards Institute (CLSI) guidelines [CLSI, 2018] to categorize the isolates into resistant, intermediate and susceptible. For downstream analysis, resistant and intermediate isolates were grouped together. Some antimicrobials had strong difference in penetration to blood and cerebrospinal fluid due to the blood–brain barrier, therefore the CLSI interpretative breakpoints for meningitis and non-meningitis cases were different. Here, meningitis cut-offs were used for penicillin (≥0.12 mg l^−1^ was categorized as resistant), cefotaxime (≥2 mg l^−1^) and ceftriaxone (≥2 mg l^−1^). Multidrug resistance (MDR) was defined as resistance to three or more classes of antimicrobials. In order to compare the carriage isolates from South Africa to the other GPS isolates collected globally, the study isolates were classified into Global Pneumococcal Sequence Clusters (GPSCs) using a genomic tool called Population Partitioning Using Nucleotide K-mers (PopPUNK) [19]. The GPSC reference database used to designate the GPSCs to each strain was downloaded from the GPS website https://www.pneumogen.net/gps/assigningGPSCs.html. A maximum-likelihood phylogenetic tree was constructed based on single nucleotide polymorphisms (SNPs), by mapping to the reference genome of *S. pneumoniae* ATCC 700669 (NCBI accession number FM211187) using FastTree [20]. An interactive visualization of the phylogenetic tree and the associated metadata was created using Microreact (https://microreact.org/project/GPS_South_Africa_carriage).

### Statistical analysis

Samples were grouped into two vaccine periods based on the date of sample collection: (1) PCV7 era: included samples collected between June 2009 and July 2011 (after the introduction of PCV7 but before PCV13), (2) PCV13 era: included samples collected between August 2011 and November 2013 (after the introduction of PCV13). *In-silico* serotypes were stratified into three groups, namely those which are covered by PCV7 (PCV7 serotypes: 4, 6B, 9V, 14, 18C, 19F and 23F), additional six serotypes covered only by PCV13 (PCV13 serotypes: 1, 3, 5, 6A, 7F, 19A), and serotypes which are not covered by either vaccine (NVTs). Further, serotypes 15B and 15C were grouped as 15B/C due to their ability to interconvert [21]. Changes in GPSC, serotype, and resistance to each antibiotic were assessed using Fisher’s exact test. Two-sided *P*-values of less than 0.05 were considered significant. Multiple testing correction was done using the Benjamini–Hochberg false discovery rate of 5 % when the number of tests is greater than ten. Statistical analysis was performed with R version 3.5.2.

## Results

### Pneumococcal collection

A total of 1825 pneumococcal carriage isolates were whole-genome sequenced and the demographic characteristics are summarized in Table 1. Overall, 55 % (995/1,825) of the isolates were collected from children aged ≤2 years old, 21 % from 3- to 5-year-old children, and 24 % from individuals aged >5 years old. In the older than 5 years age group, 40.9 % (180/440) were adults over 18 years. As the study from Soweto had a mother–child study design and mothers are usually the primary-caretakers of children in Agincourt, these studies had a higher number of adult female participants. As the Soweto survey was designed to compare the differences in the carriage prevalence of pneumococcus between participants with and without underlying HIV, a higher proportion of the isolates collected from Soweto (49.9 %; *n*=363/727) were from people living with HIV compared with those collected in Agincourt (6.8 %; *n*=26/383) [11]. Of the 1097 participants who contributed isolates in Agincourt, only 35 % (*n*=383) had a self-reported HIV status recorded. Lastly, the number of isolates collected from the PCV7 and PCV13 eras in both regions were close to 1 : 1 (Table 1).

### Changes in pneumococcal carriage serotypes

**Fig. 1.**
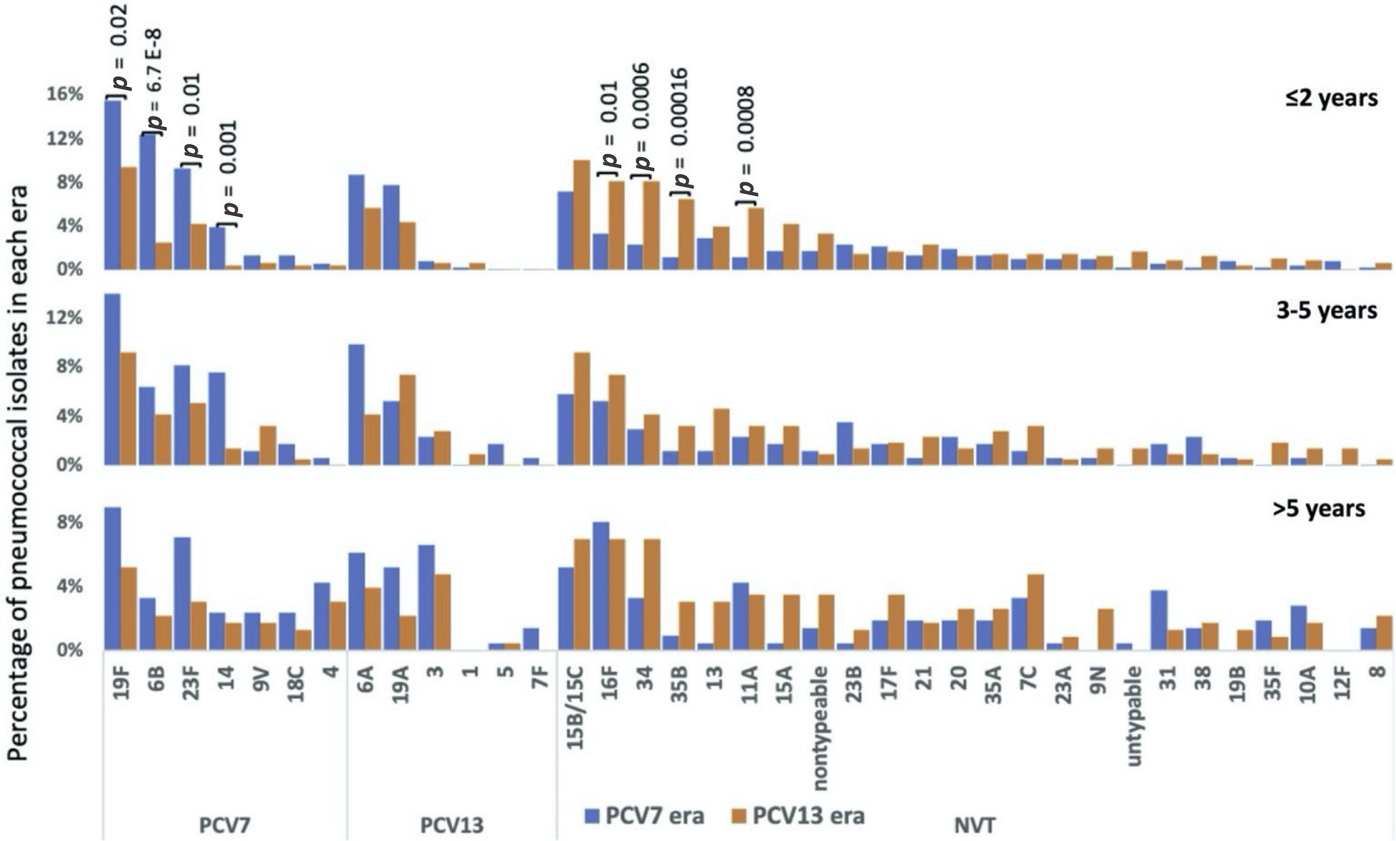
Differences in serotype distribution of carriage isolates collected in the PCV7 and PCV13 eras. The proportion of serotypes within PCV7 (blue) and PCV13 (orange) in (a) children ≤2 years of age, (b) children between 3 and 5 years of age and (c) individuals over 5 years of age. Untypable status is assigned to the strains for which no match to a pneumococcal serotype was found in the reference database.

A total of 49 serotypes were detected. Of these, the ten most prevalent serotypes were 19F (*n*=200), 15B/15C (*n*=142), 6A (*n*=120), 23F (*n*=115), 16F (*n*=114), 6B (*n*=108), 19A (*n*=102), 34 (*n*=88), 11A (*n*=61) and 35B (*n*=55), together these serotypes accounted for 58 %(1050/1825) of the whole dataset. Serotype distribution by region is shown in Table S2 and there was no significant difference in serotype prevalence between Soweto and Agincourt. The carriage prevalence of PCV7 serotypes 19F (80/516 vs. 45/479, OR: 0.6, 95 % CI: 0.37–0.85, *P*=0.02), 6B (64/516 vs. 12/479, OR: 0.2, 95 % CI: 0.09–0.35, *P*<0.005), 23F (48/516 vs. 12/479, OR: 0.3, 95 % CI: 0.12–0.49, *P*=0.01) and 14 (20/516 vs. 2/479, OR: 0.1, 95 % CI: 0.01–0.43, *P*=0.001) decreased significantly between the PCV7 and PCV13 eras among isolates collected from children ≤2 years of age (Fig. 1). The carriage prevalence of PCV7 serotypes 9V, 18C and, 4 was, however, too low (<1.5 %) in this age group for comparisons. Nevertheless, no significant changes were observed for the additional PCV13 vaccine serotypes 1, 3, 5, 6A, 7F and 19A. Further, a significant increase in four NVTs including 16F [3 % (17/516) vs. 8 % (39/479); OR: 2.6, 95 % CI: 1.41–4.97, *P*=0.01], 34[2 % (12/516) vs. 8 % (39/479); OR: 3.7, 95 % CI: 1.88–7.9, *P*=0.0006], 35B [1 % (6/516) vs. 6 % (31/479); OR: 5.9, 95 % CI: 2.38 17.38, *P*=0.00016] and 11A [1 % (6/516) vs. 6 %.(27/479); OR: 5.1, 95 % CI: 2.03–15.16, *P*=0.0008] was detected in children ≤2 years of age. A similar trend was observed for non-vaccine serotypes 15A and 21, as reported in previous studies [9–11], although the trend was not significant in this study. No significant changes in serotypes were detected in other age groups. Serotype distribution by vaccine era and GPSCs is shown in Tables S3 and S4, respectively.

### Changes in GPSC and emerging serotypes

**Fig. 2.**
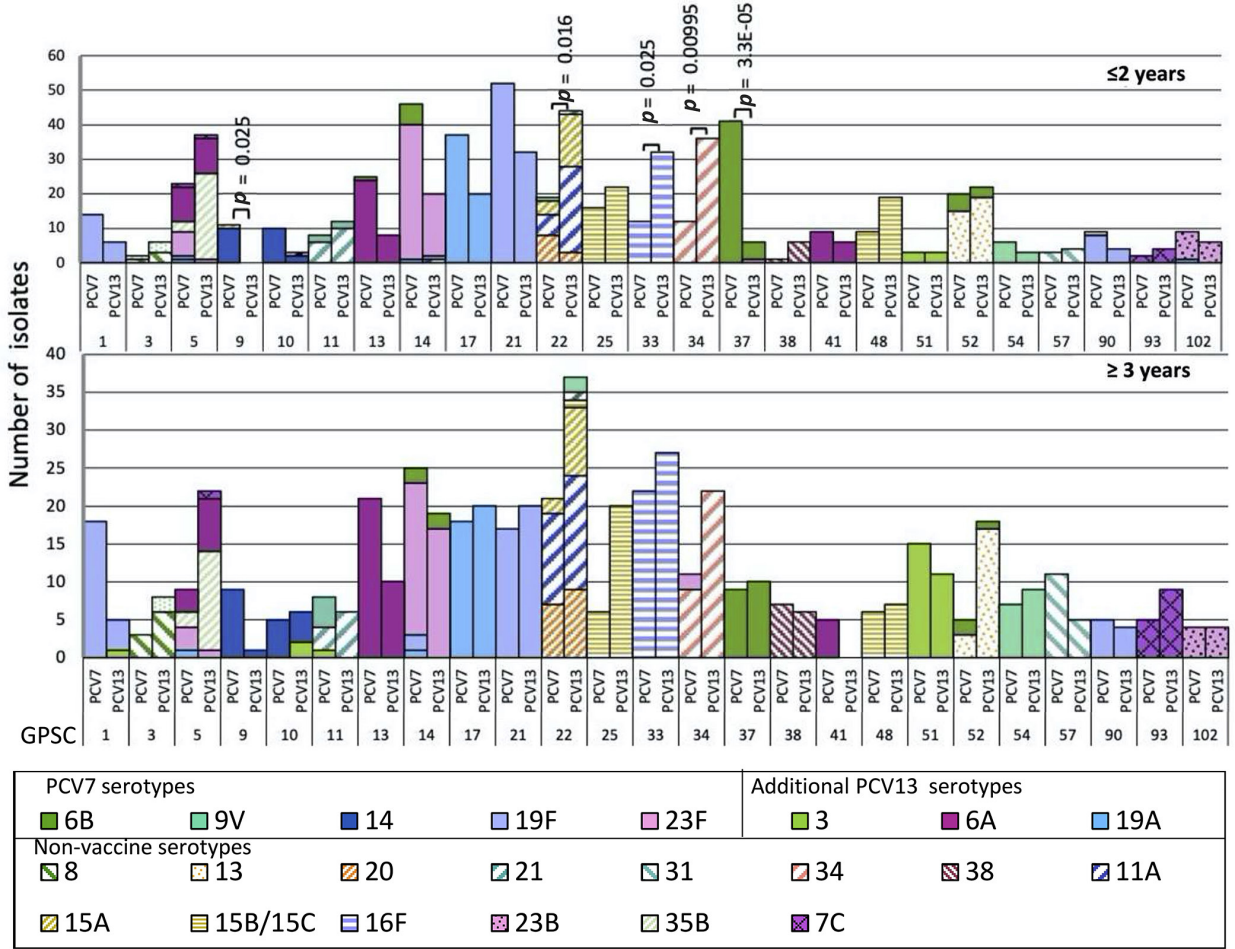
GPSC distribution with associated serotypes in carriage isolates between PCV7 and PCV13 era in South Africa. The proportion of major GPSCs within PCV7 and PCV13 in (a) children ≤2 years of age and (b) individuals over 2 years of age. *For statistical analysis, the data was stratified into two age groups: (a) children ≤2 years of age, (b) individuals over 3 years of age. Statistically significant patterns were only detected in children ≤2 years of age.

These carriage isolates represented a diverse population with 126 GPSCs detected. Notably, 105 are minor GPSCs of ≤20 isolates, each representing ~1.1 % of the dataset (Fig. S3). The ten most common GPSCs included GPSC22 (*n*=121, mainly composed of ST4984 and ST10545), GPSC21 (*n*=121, ST347), GPSC14 (*n*=110, ST6279), GPSC17 (*n*=95, ST2062), GPSC33 (*n*=93, ST4088 and ST10543), GPSC5 (*n*=91, ST361, ST1447, ST172), GPSC34 (*n*=81, ST7067), GPSC37 (*n*=66, ST4929, ST2909, ST2421), GPSC52 (*n*=65, ST5647), and GPSC13 (*n*=64, ST5073, ST2285), which together accounted for 50 % of the whole dataset. All GPSC21 (serotype 19F), GPSC17 (serotype 19A), GPSC37 (serotype 6B, 23F) and GPSC13 (serotype 6A, 6B) isolates were VT whereas all GPSC33 (serotype 16F) isolates were NVT. In GPSC22 (serotype 11A, 15A, 20, 9V, 35A, 15B/C), GPSC34 (serotype 34 and 23F) and GPSC52 (serotype 13 and 6B)>80 % of the isolates were NVT, and 97 % of GPSC14 (serotype 23F, 6B, 19F, 19A, 11A) isolates were VT. Interestingly, GPSC5 had an almost even mix of NVT (51%, serotypes 35B and 7C) and VT (49%, serotypes 6A, 23F, 19F and 19A) (Fig. 2).

**Fig. 3.**
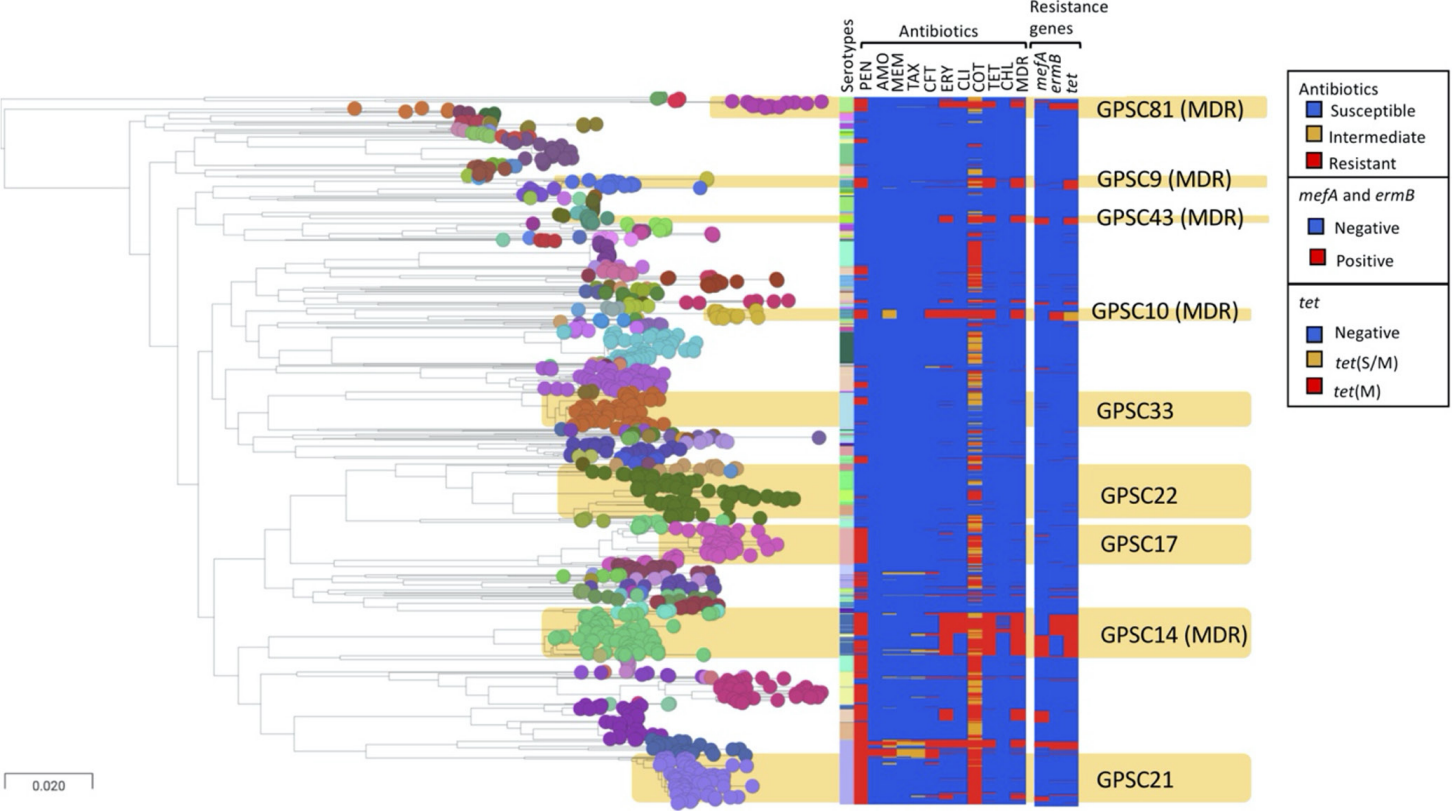
Population structure, serotype and resistance profile of carriage isolates from South Africa. The nodes of the tree indicate GPSCs. *In silico* serotype, antibiotic resistance and selected resistance markers are included in the metablock. Penicillin resistance was predicted based on the *pbp*1a, *pbp*2b, *pbp*2x sequences; tetracycline and erythromycin resistance were predicted based on the presence of *tet*(M), *tet*(O) and *tet*(S/M), and *erm*(B) and *mef*(A), respectively. Cotrimoxazole resistance was predicted based on the presence of mutation I100L in *fol*A and any indel within amino acid residue 56–67 in *fol*P while presence of either mutation predicted to be cotrimoxazole-intermediate. PEN, penicillin; AMO, amoxicillin; MER, meropenem; TAX, cefotaxime; CFT, ceftriaxone; CFX, cefuroxime; ERY, erythromycin; CLI, clindamycin; TET, tetracycline; DOX, doxycycline; CHL, chloramphenicol; MDR, multidrug resistant. This figure can be visualized at https://microreact.org/project/GPS_South_Africa_carriage.

Among children ≤2 years of age, a significant decrease was observed in the prevalence of GPSC9 and GPSC37 lineages that mainly expressed serotype 14 and 6B, respectively (Fig. 2). Significant increases in proportion were detected between the PCV7 and PCV13 eras in three NVT pneumococcal lineages: GPSC22 (serotypes 11A, 15A, 20, 35A, and 15B/C) increased from 3.7 % (19/516) to 9.2 % (44/479) (OR: 3.2, 95 % CI: 1.78–5.84, *P*=0. 016), GPSC33 (serotype 16F) from 2.3 % (12/516) to 6.7 % (32/479) (OR: 3.0, 95 % CI: 1.48–6.48, *P*=0.025), and GPSC34 (serotype 34) from 2.3 % (12/516) to 7.5 % (36/479) (OR: 3.4, 95 % CI: 1.71–7.29, *P*=0.00995) (Fig. 2). The increase in these GPSCs was the main driver behind the increase in prevalence of the carriage NVTs 11A, 16F, and 34, in this age group (Fig. S4). The increase in serotype 35B was mainly reflected by changes in the composition of GPSC5 in the PCV13 era, which was largely composed of vaccine serotypes 6A and 23F in the PCV7 era (Figs S1 and S4). The composition of serotypes in each GPSC is similar between the two age groups (≤2 years old and ≥3 years old) (Fig. 2). No significant changes in GPSC were detected in the latter age group between isolates collected in the PCV7 and PCV13 eras. Comparison of GPSCs between the two regions showed that there was higher proportion of GPSC1 (4.4 % in Soweto vs. 1 % in Agincourt; *P*<0.05) and GPSC10 (2.5 % in vs. 0.5 %; *P*=0.02) in Soweto as compared to Agincourt (Table S5). In contrast, GPSC21 (8.8 % in Agincourt vs. 3.4 % in Soweto; *P*<0.05) and GPSC37 (4.7 % vs. 1.9 %; *P*=0.03) were more prevalent in Agincourt (Table S5). Comparison of GPSCs between individuals with and without underlying HIV did not show any significant difference. The detailed distribution of GPSCs by serotype, and GPSCs by vaccine era, are listed in Tables S4 and S6. A phylogenetic tree with the associated metadata is shown in Fig. 3.

### Changes in antimicrobial resistance in pneumococcal carriage isolates between the PCV7 and PCV13 period

A significant decrease in the proportion of antimicrobial resistance among carriage isolates from children ≤2 years of age, was detected from the PCV7 to the PCV13 era (Table 2). Further, resistance against cefuroxime also decreased significantly in isolates from children between the ages of 3 and 5 years (*P*=0.034, OR: 0.3, 95 % CI: 0.12–0.67). No significant changes were observed in predicted antimicrobial resistance among isolates from individuals >5 years of age. Non-susceptibility to ceftriaxone was significantly lower among GPSC14 isolates (21.13 %–0 %; OR: 0, 95 % CI: 0.00–0.43, *P*=0.02) in the PCV13 era as compared to the PCV7 era. Ceftriaxone non-susceptible isolates expressing vaccine serotypes 6B and 23F were mainly grouped into two clusters within GPSC14 in the phylogenetic tree. These clusters were not detected in the PCV13 era. (Fig. S2; https://microreact.org/project/GPS_South_Africa_carriage/f0511fbf). Overall, VTs were more closely associated with antibiotic resistance than NVTs (Table S7).

**Table 2.** Changes in proportion of resistance to 17 antibiotics in pneumococcal carriage isolates stratified by age

Changes in resistance in pneumococcal isolates in the PCV7 era and PCV13 era in different age groups % (*n*)									
**Antibiotics**	**Children ≤2 years of age**			**Children between 3 to 5 years**			**Adults over 5 years**		
	**PCV7 era (** ***N*** **=516)**	**PCV13 era (** ***N*** **=479)**	***P*-value**	**PCV7 era (** ***N*** **=172)**	**PCV13 era (** ***N*** **=218)**	***P*** **-value**	**PCV7 era (** ***N*** **=211)**	**PCV13 era (** ***N*** **=229)**	***P*** **-value**
Penicillin	55.5 % (286)	41.8 % (200)	<0.001	46.6 % (80)	41.3 % (90)	0.56	37 % (78)	30.3 % (69)	0.32
Amoxicillin	2.6 % (13)	0.7 % (3)	0.04	4.7 % (8)	0.5 % (1)	0.06	2.4 % (5)	0.5 % (1)	0.32
Meropenem	7.4 % (38)	2.3 % (11)	<0.001	9.9 % (17)	3.7 % (8)	0.08	6.2 % (13)	2.2 % (5)	0.32
Cefotaxime	4.1 % (21)	1.1 % (5)	0.006	5.3 % (9)	2.3 % (5)	0.38	3.8 % (8)	0.9 % (2)	0.32
Ceftriaxone	6.4 % (33)	1.5 % (7)	<0.001	8.2 % (14)	2.3 % (5)	0.06	4.3 % (9)	1.8 % (4)	0.32
Cefuroxime	9.9 % (51)	3.2 % (15)	<0.001	14 % (24)	4.6 % (10)	0.035	7.2 % (15)	3.6 % (8)	0.32
Erythromycin	20 % (103)	12.2 % (58)	0.002	17.5 % (30)	12.9 % (28)	0.50	15.7 % (33)	13.6 % (31)	1.00
Clindamycin	10.7 % (55)	5.7 % (27)	0.009	9.4 % (16)	4.6 % (10)	0.23	9.5 % (20)	5.3 % (12)	0.29
Quinupristin-dalfopristin (Synercid)	0	0	1.00	0	0	1.00	0	0	1.00
Linezolid	0	0	1.00	0	0	1.00	0	0	1.00
Co-trimoxazole	77.2 % (398)	71 % (340)	0.049	66.3 % (114)	68.9 % (150)	1.00	58.3 % (123)	57.3 % (131)	1.00
Tetracycline	18 % (93)	10.2 % (49)	0.002	19.8 % (34)	12.8 % (28)	0.18	15.2 % (32)	10.5 % (24)	0.32
Doxycycline	18 % (93)	10.2 % (49)	0.002	19.8 % (34)	12.8 % (28)	0.18	15.2 % (32)	10.5 % (24)	0.32
Levofloxacin	0	0	1.00	0	0	1.00	0	0	1.00
Chloramphenicol	2.2 % (11)	2.1 % (10)	1.00	1.8 % (3)	1.9 % (4)	1.00	1 % (2)	0.5 % (1)	1.00
Rifampin	0	0	1.00	0	0	1.00	0	0	1.00
Vancomycin	0	0	1.00	0	0	1.00	0	0	1.00

There was one isolate with no predicted output for the beta-lactams from individuals over 5 years in the PCV13 era. This isolate was taken out of the analysis for calculating the percentages and *P*-values for beta-lactams. Resistant and intermediate isolates were grouped together into the resistant category for this comparison.

Among NVT isolates from children aged 3–5 years, there was a significant increase in resistance against penicillin (4.3 % vs. 21.7 %, OR: 6.1, 95 % CI: 1.79–32.66, *P*=0.02), in the PCV13 era compared to the PCV7 era (Table S8). A similar increasing trend was observed among isolates from children ≤2 years of age, although the trend was not statistically significant (OR: 2.0, 95 % CI: 1.22–3.31, *P*=0.08). Among NVT isolates from children <5 years of age, GPSC5 (28%), GPSC48 (21 %), GPSC33 (10 %), GPSC81 (6 %) and GPSC148 (5 %) were the major contributors of penicillin-resistant isolates in the PCV13 era. These GPSCs remained the major contributors of penicillin resistance when individuals over 5 years were added to the data as well.

## Discussion

This study reports the changing population structure of pneumococcal carriage isolates collected in two regions in South Africa that have a high HIV disease burden, Agincourt (rural) and Soweto (urban), in the PCV7 and PCV13 eras. One of the major findings of this study was the decrease in carriage rates of four VTs: 19F, 23F, 6B and 14, and corresponding increase in NVTs 16F, 34, 35B and 11A, among children ≤2 years of age in the PCV13 era. These findings are in-line with previous studies from South Africa [9–11]. The slight differences between the findings of this study and the previous studies could be due to different grouping schemes for vaccine periods and age groups. As the current dataset is a subset of the isolates from the previous survey, we observed a similar trend of high residual colonization by VTs 19F and 23F in the PCV13 era [9–11].

Based on a global dataset of pneumococcal isolates (*n*=13 454) that were previously reported, and data from the PubMLST database (https://pubmlst.org/bigsdb?db=pubmlst_spneumoniae_isolates&page=profiles), most of the top ten pneumococcal lineages found in this study are endemic to Africa, except for GPSC13, GPSC5, GPSC14 and GPSC37 (Table S9) [22]. All of these prevalent lineages are also found to cause IPD in South Africa [23]. Pneumococcal lineages that mainly consisted of VT such as GPSC21 (serotype 19F), 14 (23F), 17 (19A), 37 (6B) and 13 (6A), and were found to significantly decrease in the disease-causing population, did not decrease significantly in the carriage population, except for GPSC37. GPSC21 and GPSC14 were the major pneumococcal lineages that accounted for the residual vaccine serotypes 19F and 23F reported in the recent study from Agincourt [9].

GPSC21 is mainly found in the southern part of Africa (e.g. Malawi and Mozambique, https://microreact.org/project/gpsGPSC21). In contrast, GPSC14, which accounts for >90 % of serotype 23F in the PCV13 era globally, can be found across continents. A global phylogeny of GPSC14 showed that the South African isolates are clustered into two deep branched clades, suggesting the divergence is not recent (https://microreact.org/project/gpsGPSC14) [22]. The two clades differ in erythromycin-resistance genes carried; one carries *mefA* and the other *ermB*. Most isolates of GPSC14 expressed serotype 23F, except for a clade of both carriage and disease isolates from South Africa which expressed serotype 6B.

Notably, GPSC21 and GPSC14, which were responsible for the residual VTs, were penicillin-resistant, and GPSC14 is a MDR lineage with additional resistance to erythromycin, cotrimoxazole and tetracycline. Interestingly, we found that Soweto, an urban city in close proximity of Johannesburg, had a higher proportion of the globally spreading lineages GPSC1 and GPSC10, as compared to the rural Agincourt. While Agincourt had a higher proportion of the lineages GPSC21 and GPSC37, which are prevalent in the southern part of Africa (Table S5).

Similarly to what has been previously reported among the disease-causing pneumococcal population [22], we observed the expansion of non-vaccine serotype 35B within GPSC5, and a significant increase in the GPSC33 lineage expressing serotype 16F among isolates from children aged 2 years and under [23]. The GPSC5 lineage expressing 15 serotypes (five VTs and ten NVTs) has reportedly spread across the world [12]. Serotype 35D, which has been associated with higher invasive disease potential compared to serotype 35B, was not found in this study, but was observed in the disease-causing pneumococcal population in South Africa [24]. All serotype 35B/D variants within GPSC5 in the GPS dataset are clustered together in the global lineage phylogeny (https://microreact.org/project/gpsGPSC5). They are mainly found in South Africa, Malawi and Mozambique, indicating a clonal expansion of this serotype variant in Africa, followed by a single capsular switching event. GPSC5 was also observed elsewhere but mainly expressing serotypes other than 35B/D. GPSC33 was only found in the Southern part of Africa with most isolates expressing serotype 16F (https://microreact.org/project/gpsGPSC33).

Reductions in resistance to clinically important antibiotics in the carriage population overall were observed in the PCV13 era, indicating that PCVs have a positive impact in reducing antibiotic resistance through a direct reduction of antibiotic-resistant VTs, and perhaps secondarily through reduction in febrile illness that often requires antibiotic use [25]. Increasing penicillin resistance among NVT pneumococci is mainly due to clonal expansion of pneumococcal lineages that already exhibited penicillin resistance in the PCV7 era, rather than the recent acquisition of antibiotic resistance [23]. Such expansion could be a result of both vaccine- and antibiotic-selective pressure and requires close monitoring.

This study is limited in that we did not have information on the HIV status of all the participants, with most of the participants missing data on HIV status being from Agincourt. Colonization rates of different serotypes vary between individuals with and without HIV; however, due to the incomplete data on HIV status, we could not assess the potential differences in the prevalence of serotypes, lineages, and resistance profiles between these cohorts.

In conclusion, this study reports the changes in carriage epidemiology in South Africa, before and after the introduction of PCV13, with most of the carriage isolates from South Africa belonging to pneumococcal lineages which are endemic to Africa (GPSC22, GPSC21, GPSC17, GPSC33, GPSC34 and GPSC52). Of these endemic lineages, GPSC22, GPSC33, GPSC34 were the main drivers of increases in carriage of NVT 11A, 16F and 34, respectively. The increase in serotype 35B was mainly due to the globally spreading pneumococcal lineage GPSC5. While the introduction of PCVs has resulted in decreased levels of resistance among pneumococcal carriage isolates, an increase in penicillin resistance in NVTs was observed in isolates from children between 3 and 5 years of age, highlighting the expansion of penicillin-resistant clones associated with NVTs in the PCV13 era.

## Supplementary Data

Supplementary material 1

Supplementary material 2

Supplementary material 3
