# Supplementary material for: Population genomics of pneumococcal carriage in South Africa following the introduction of the 13-valent pneumococcal conjugate vaccine (PCV13) immunization
Source: Microb Genom. 2022 Jun 23;8(6):mgen000831. doi: 10.1099/mgen.0.000831 (PMC9455715; doi:10.1099/mgen.0.000831)
Supplement: Supplementary material 1 [file mgen-8-831-s001.pdf]

Table S1. Complete dataset of the study
(attached as an excel file)

Table S2. Distribution of serotypes among the study regions.

**Serotypes**	**Agincourt n(%)**	**Soweto n(%)**	**Total n(%)**	**p-value**
**PCV7**	347(31.7%)	194(26.7%)	541(29.7%)	
19F	130(11.9%)	70(9.7%)	200(11%)	0.56
23F	70(6.4%)	45(6.2%)	115(6.4%)	1
6B	77(7.1%)	31(4.3%)	108(6%)	0.23
14	25(2.3%)	22(3.1%)	47(2.6%)	0.74
9V	17(1.6%)	11(1.6%)	28(1.6%)	1
4	17(1.6%)	5(0.7%)	22(1.3%)	0.56
18C	11(1.1%)	10(1.4%)	21(1.2%)	0.74
**PCV13**	163(14.9%)	116(16%)	279(15.3%)	
6A	77(7.1%)	43(6%)	120(6.6%)	0.74
19A	55(5.1%)	47(6.5%)	102(5.6%)	0.66
3	28(2.6%)	14(2%)	42(2.4%)	0.74
1	1(0.1%)	5(0.7%)	6(0.4%)	0.29
5	2(0.2%)	3(0.5%)	5(0.3%)	0.74
7F	(0%)	4(0.6%)	4(0.3%)	0.25
**NVT**	587(53.6%)	418(57.5%)	1005(55.1%)	
15B/15C	88(8.1%)	54(7.5%)	142(7.8%)	0.84

16F	62(5.7%)	52(7.2%)	114(6.3%)	0.66
34	57(5.2%)	31(4.3%)	88(4.9%)	0.74
11A	31(2.9%)	30(4.2%)	61(3.4%)	0.56
35B	36(3.3%)	19(2.7%)	55(3.1%)	0.74
13	35(3.2%)	19(2.7%)	54(3%)	0.77
15A	19(1.8%)	29(4%)	48(2.7%)	0.11
Nontypable	23(2.1%)	17(2.4%)	40(2.2%)	0.91
7C	26(2.4%)	13(1.8%)	39(2.2%)	0.74
17F	19(1.8%)	19(2.7%)	38(2.1%)	0.71
20	16(1.5%)	17(2.4%)	33(1.9%)	0.66
35A	17(1.6%)	16(2.2%)	33(1.9%)	0.74
23B	24(2.2%)	8(1.1%)	32(1.8%)	0.56
21	19(1.8%)	13(1.8%)	32(1.8%)	1
31	15(1.4%)	8(1.1%)	23(1.3%)	0.84
9N	15(1.4%)	6(0.9%)	21(1.2%)	0.74
10A	12(1.1%)	8(1.1%)	20(1.1%)	1
38	11(1.1%)	9(1.3%)	20(1.1%)	0.84
23A	7(0.7%)	10(1.4%)	17(1%)	0.56
35F	8(0.8%)	8(1.1%)	16(0.9%)	0.74
8	8(0.8%)	5(0.7%)	13(0.8%)	1

untypable	11(1.1%)	2(0.3%)	13(0.8%)	0.56
19B	8(0.8%)	3(0.5%)	11(0.7%)	0.75
12F	3(0.3%)	4(0.6%)	7(0.4%)	0.74
22F	1(0.1%)	6(0.9%)	7(0.4%)	0.23
33D	1(0.1%)	5(0.7%)	6(0.4%)	0.29
6C	1(0.1%)	3(0.5%)	4(0.3%)	0.74
37	3(0.3%)	(0%)	3(0.2%)	0.74
10B	3(0.3%)	(0%)	3(0.2%)	0.74
22A	1(0.1%)	1(0.2%)	2(0.2%)	1
18A	1(0.1%)	1(0.2%)	2(0.2%)	1
serogroup 24	2(0.2%)	(0%)	2(0.2%)	0.74
28F	1(0.1%)	1(0.2%)	2(0.2%)	1
29	2(0.2%)	(0%)	2(0.2%)	0.74
46	1(0.1%)	(0%)	1(0.1%)	1
10F	(0%)	1(0.2%)	1(0.1%)	0.74
**Total**	**1097**	**728**	**1825**	

No significant differences in serotypes distribution was detected between the two regions. Untypable status is assigned to the strains for which no match to a pneumococcal serotype was found in the reference database whereas nontypeable strains are the ones which show no evidence of capsular expression. Nontypeable strains may have no or some of the cps locus genes.

Table S3. Distribution of serotypes between PCV7 and PCV13 era

Serotypes	PCV7 era<br>%(n)	PCV13 era<br>%(n)	Total<br>%(n)

**PCV7**	362(40.3%)	179(19.4%)	541(29.7%)
19F	123(13.7%)	77(8.4%)	200(11%)
23F	77(8.6%)	38(4.2%)	115(6.4%)
6B	82(9.2%)	26(2.9%)	108(6%)
14	38(4.3%)	9(1%)	47(2.6%)
9V	14(1.6%)	14(1.6%)	28(1.6%)
4	13(1.5%)	9(1%)	22(1.3%)
18C	15(1.7%)	6(0.7%)	21(1.2%)
**PCV13 additional**	166(18.5%)	113(12.3%)	279(15.3%)
6A	75(8.4%)	45(4.9%)	120(6.6%)
19A	60(6.7%)	42(4.6%)	102(5.6%)
3	22(2.5%)	20(2.2%)	42(2.4%)
1	1(0.2%)	5(0.6%)	6(0.4%)
5	4(0.5%)	1(0.2%)	5(0.3%)
7F	4(0.5%)	(0%)	4(0.3%)
**NVT**	371(41.3%)	634(68.5%)	1005(55.1%)
15B/15C	58(6.5%)	84(9.1%)	142(7.8%)
16F	43(4.8%)	71(7.7%)	114(6.3%)
34	24(2.7%)	64(7%)	88(4.9%)
11A	19(2.2%)	42(4.6%)	61(3.4%)
35B	10(1.2%)	45(4.9%)	55(3.1%)
13	18(2.1%)	36(3.9%)	54(3%)

15A	13(1.5%)	35(3.8%)	48(2.7%)
Nontypable	14(1.6%)	26(2.9%)	40(2.2%)
7C	14(1.6%)	25(2.7%)	39(2.2%)
17F	18(2.1%)	20(2.2%)	38(2.1%)
20	18(2.1%)	15(1.7%)	33(1.9%)
35A	14(1.6%)	19(2.1%)	33(1.9%)
23B	19(2.2%)	13(1.5%)	32(1.8%)
21	12(1.4%)	20(2.2%)	32(1.8%)
31	14(1.6%)	9(1%)	23(1.3%)
9N	6(0.7%)	15(1.7%)	21(1.2%)
10A	9(1.1%)	11(1.2%)	20(1.1%)
38	8(0.9%)	12(1.3%)	20(1.1%)
23A	7(0.8%)	10(1.1%)	17(1%)
35F	5(0.6%)	11(1.2%)	16(0.9%)
8	4(0.5%)	9(1%)	13(0.8%)
untypable	2(0.3%)	11(1.2%)	13(0.8%)
19B	5(0.6%)	6(0.7%)	11(0.7%)
12F	4(0.5%)	3(0.4%)	7(0.4%)
22F	2(0.3%)	5(0.6%)	7(0.4%)
33D	3(0.4%)	3(0.4%)	6(0.4%)
6C	2(0.3%)	2(0.3%)	4(0.3%)
37	1(0.2%)	2(0.3%)	3(0.2%)

10B	(0%)	3(0.4%)	3(0.2%)
22A	(0%)	2(0.3%)	2(0.2%)
18A	1(0.2%)	1(0.2%)	2(0.2%)
serogroup 24	1(0.2%)	1(0.2%)	2(0.2%)
28F	1(0.2%)	1(0.2%)	2(0.2%)
29	1(0.2%)	1(0.2%)	2(0.2%)
46	1(0.2%)	(0%)	1(0.1%)
10F	(0%)	1(0.2%)	1(0.1%)
**Total**	**899**	**926**	**1825**

Untypable status is assigned to the strains for which no match to a pneumococcal serotype was found in the reference database whereas nontypeable strains are the ones which show no evidence of capsular expression. Nontypeable strains may have no or some of the cps locus genes.

Table S4. Distribution of GPSCs within each serotype
(attached as an excel file)

Table S5. Distribution of GPSCs between Agincourt and Soweto

**GPSC**	**Agincourt n(%)**	**Soweto n(%)**	**p-value**
1	11 (1%)	32 (4.4%)	<0.005
21	96 (8.8%)	25 (3.4%)	<0.005
10	6 (0.5%)	18 (2.5%)	0.02
37	52 (4.7%)	14 (1.9%)	0.03
22	57 (5.2%)	64 (8.8%)	0.06
79	16 (1.5%)	2 (0.3%)	0.22
133	16 (1.5%)	2 (0.3%)	0.22
46	0 (0%)	4 (0.5%)	0.29
125	15 (1.4%)	2 (0.3%)	0.29
32	0 (0%)	4 (0.5%)	0.29
61	6 (0.5%)	12 (1.6%)	0.29
2	1 (0.1%)	5 (0.7%)	0.39
5	64 (5.8%)	27 (3.7%)	0.43
200	0 (0%)	3 (0.4%)	0.5

305	0 (0%)	3 (0.4%)	0.5
25	31 (2.8%)	33 (4.5%)	0.51
7	4 (0.4%)	8 (1.1%)	0.53
211	1 (0.1%)	4 (0.5%)	0.57
26	0 (0%)	2 (0.3%)	0.67
48	30 (2.7%)	11 (1.5%)	0.67
88	4 (0.4%)	0 (0%)	0.67
17	50 (4.6%)	45 (6.2%)	0.67
201	5 (0.5%)	0 (0%)	0.67
23	0 (0%)	2 (0.3%)	0.67
306	5 (0.5%)	0 (0%)	0.67
34	55 (5%)	26 (3.6%)	0.67
142	0 (0%)	2 (0.3%)	0.67
207	2 (0.2%)	5 (0.7%)	0.67
210	5 (0.5%)	0 (0%)	0.67
233	0 (0%)	2 (0.3%)	0.67
9	16 (1.5%)	5 (0.7%)	0.71
14	60 (5.5%)	50 (6.9%)	0.73
168	0 (0%)	1 (0.1%)	0.73
102	17 (1.5%)	6 (0.8%)	0.73
192	1 (0.1%)	3 (0.4%)	0.73
77	9 (0.8%)	2 (0.3%)	0.73
18	3 (0.3%)	0 (0%)	0.73
159	7 (0.6%)	2 (0.3%)	0.73
238	0 (0%)	1 (0.1%)	0.73
129	0 (0%)	1 (0.1%)	0.73
258	8 (0.7%)	2 (0.3%)	0.73
3	9 (0.8%)	10 (1.4%)	0.73
11	17 (1.5%)	17 (2.3%)	0.73
514	0 (0%)	1 (0.1%)	0.73
504	0 (0%)	1 (0.1%)	0.73
123	3 (0.3%)	0 (0%)	0.73
355	0 (0%)	1 (0.1%)	0.73
299	0 (0%)	1 (0.1%)	0.73
163	0 (0%)	1 (0.1%)	0.73
179	3 (0.3%)	5 (0.7%)	0.73
246	6 (0.5%)	1 (0.1%)	0.73
512	0 (0%)	1 (0.1%)	0.73
505	0 (0%)	1 (0.1%)	0.73

515	0 (0%)	1 (0.1%)	0.73
304	0 (0%)	1 (0.1%)	0.73
259	1 (0.1%)	3 (0.4%)	0.73
178	1 (0.1%)	3 (0.4%)	0.73
93	15 (1.4%)	5 (0.7%)	0.73
137	1 (0.1%)	3 (0.4%)	0.73
16	0 (0%)	1 (0.1%)	0.73
51	22 (2%)	10 (1.4%)	0.73
242	3 (0.3%)	0 (0%)	0.73
8	2 (0.2%)	3 (0.4%)	0.73
171	3 (0.3%)	0 (0%)	0.73
83	0 (0%)	1 (0.1%)	0.73
513	0 (0%)	1 (0.1%)	0.73
253	0 (0%)	1 (0.1%)	0.73
330	0 (0%)	1 (0.1%)	0.73
49	11 (1%)	4 (0.5%)	0.78
160	3 (0.3%)	4 (0.5%)	0.8
278	2 (0.2%)	0 (0%)	0.84
90	15 (1.4%)	7 (1%)	0.84
184	2 (0.2%)	0 (0%)	0.84
145	2 (0.2%)	0 (0%)	0.84
114	4 (0.4%)	5 (0.7%)	0.84
199	4 (0.4%)	5 (0.7%)	0.84
301	2 (0.2%)	0 (0%)	0.84
206	2 (0.2%)	0 (0%)	0.84
255	1 (0.1%)	2 (0.3%)	0.88
30	6 (0.5%)	6 (0.8%)	0.88
91	1 (0.1%)	2 (0.3%)	0.88
38	11 (1%)	9 (1.2%)	0.94
67	2 (0.2%)	2 (0.3%)	0.94
4	4 (0.4%)	1 (0.1%)	0.94
243	2 (0.2%)	2 (0.3%)	0.94
126	9 (0.8%)	8 (1.1%)	0.94
12	2 (0.2%)	2 (0.3%)	0.94
54	14 (1.3%)	11 (1.5%)	0.95
60	3 (0.3%)	3 (0.4%)	0.95
57	15 (1.4%)	8 (1.1%)	0.95
70	3 (0.3%)	3 (0.4%)	0.95
52	41 (3.7%)	24 (3.3%)	0.95

148	5 (0.5%)	2 (0.3%)	0.96
40	9 (0.8%)	5 (0.7%)	1
33	56 (5.1%)	37 (5.1%)	1
41	12 (1.1%)	8 (1.1%)	1
13	38 (3.5%)	26 (3.6%)	1
47	1 (0.1%)	0 (0%)	1
81	10 (0.9%)	8 (1.1%)	1
470	1 (0.1%)	0 (0%)	1
254	2 (0.2%)	1 (0.1%)	1
45	1 (0.1%)	0 (0%)	1
218	1 (0.1%)	0 (0%)	1
35	4 (0.4%)	3 (0.4%)	1
194	7 (0.6%)	3 (0.4%)	1
358	1 (0.1%)	0 (0%)	1
466	1 (0.1%)	0 (0%)	1
56	3 (0.3%)	2 (0.3%)	1
143	1 (0.1%)	0 (0%)	1
68	8 (0.7%)	4 (0.5%)	1
95	1 (0.1%)	0 (0%)	1
276	3 (0.3%)	1 (0.1%)	1
469	1 (0.1%)	0 (0%)	1
468	1 (0.1%)	0 (0%)	1
116	2 (0.2%)	1 (0.1%)	1
169	3 (0.3%)	2 (0.3%)	1
506	1 (0.1%)	1 (0.1%)	1
43	11 (1%)	7 (1%)	1
467	1 (0.1%)	0 (0%)	1
473	1 (0.1%)	0 (0%)	1
92	6 (0.5%)	5 (0.7%)	1
329	1 (0.1%)	0 (0%)	1
280	1 (0.1%)	0 (0%)	1
20	1 (0.1%)	0 (0%)	1
170	1 (0.1%)	0 (0%)	1
471	1 (0.1%)	0 (0%)	1

Table S6. Distribution of GPSCs between PCV7 and PCV13 era

**GPSCs**	**PCV7 era n(%)**	**PCV13 era n(%)**	**Total n(%)**
**22**	40(4.5%)	81(8.8%)	121(6.7%)

**21**	69(7.7%)	52(5.7%)	121(6.7%)
**14**	71(7.9%)	39(4.3%)	110(6.1%)
**17**	55(6.2%)	40(4.4%)	95(5.3%)
**33**	34(3.8%)	59(6.4%)	93(5.1%)
**5**	32(3.6%)	59(6.4%)	91(5%)
**34**	23(2.6%)	58(6.3%)	81(4.5%)
**37**	50(5.6%)	16(1.8%)	66(3.7%)
**52**	25(2.8%)	40(4.4%)	65(3.6%)
**13**	46(5.2%)	18(2%)	64(3.6%)
**25**	22(2.5%)	42(4.6%)	64(3.6%)
**1**	32(3.6%)	11(1.2%)	43(2.4%)
**48**	15(1.7%)	26(2.9%)	41(2.3%)
**11**	16(1.8%)	18(2%)	34(1.9%)
**51**	18(2.1%)	14(1.6%)	32(1.8%)
**54**	13(1.5%)	12(1.3%)	25(1.4%)
**10**	15(1.7%)	9(1%)	24(1.4%)
**57**	14(1.6%)	9(1%)	23(1.3%)
**102**	13(1.5%)	10(1.1%)	23(1.3%)
**90**	14(1.6%)	8(0.9%)	22(1.3%)
**9**	20(2.3%)	1(0.2%)	21(1.2%)
**41**	14(1.6%)	6(0.7%)	20(1.1%)
**93**	7(0.8%)	13(1.5%)	20(1.1%)
**38**	8(0.9%)	12(1.3%)	20(1.1%)

**3**	5(0.6%)	14(1.6%)	19(1.1%)
**61**	8(0.9%)	10(1.1%)	18(1%)
**79**	10(1.2%)	8(0.9%)	18(1%)
**81**	5(0.6%)	13(1.5%)	18(1%)
**43**	7(0.8%)	11(1.2%)	18(1%)
**133**	11(1.3%)	7(0.8%)	18(1%)
**125**	6(0.7%)	11(1.2%)	17(1%)
**126**	6(0.7%)	11(1.2%)	17(1%)
**49**	9(1.1%)	6(0.7%)	15(0.9%)
**40**	4(0.5%)	10(1.1%)	14(0.8%)
**68**	10(1.2%)	2(0.3%)	12(0.7%)
**7**	4(0.5%)	8(0.9%)	12(0.7%)
**30**	5(0.6%)	7(0.8%)	12(0.7%)
**77**	10(1.2%)	1(0.2%)	11(0.7%)
**92**	1(0.2%)	10(1.1%)	11(0.7%)
**194**	7(0.8%)	3(0.4%)	10(0.6%)
**258**	6(0.7%)	4(0.5%)	10(0.6%)
**199**	7(0.8%)	2(0.3%)	9(0.5%)
**114**	4(0.5%)	5(0.6%)	9(0.5%)
**159**	5(0.6%)	4(0.5%)	9(0.5%)
**179**	5(0.6%)	3(0.4%)	8(0.5%)
**207**	3(0.4%)	4(0.5%)	7(0.4%)
**160**	4(0.5%)	3(0.4%)	7(0.4%)

**246**	6(0.7%)	1(0.2%)	7(0.4%)
**35**	3(0.4%)	4(0.5%)	7(0.4%)
**148**	1(0.2%)	6(0.7%)	7(0.4%)
**60**	3(0.4%)	3(0.4%)	6(0.4%)
**2**	1(0.2%)	5(0.6%)	6(0.4%)
**70**	3(0.4%)	3(0.4%)	6(0.4%)
**56**	3(0.4%)	2(0.3%)	5(0.3%)
**210**	5(0.6%)	(0%)	5(0.3%)
**306**	(0%)	5(0.6%)	5(0.3%)
**211**	(0%)	5(0.6%)	5(0.3%)
**4**	1(0.2%)	4(0.5%)	5(0.3%)
**8**	4(0.5%)	1(0.2%)	5(0.3%)
**169**	2(0.3%)	3(0.4%)	5(0.3%)
**201**	1(0.2%)	4(0.5%)	5(0.3%)
**12**	2(0.3%)	2(0.3%)	4(0.3%)
**192**	2(0.3%)	2(0.3%)	4(0.3%)
**67**	2(0.3%)	2(0.3%)	4(0.3%)
**88**	(0%)	4(0.5%)	4(0.3%)
**276**	2(0.3%)	2(0.3%)	4(0.3%)
**137**	1(0.2%)	3(0.4%)	4(0.3%)
**243**	(0%)	4(0.5%)	4(0.3%)
**46**	2(0.3%)	2(0.3%)	4(0.3%)
**259**	(0%)	4(0.5%)	4(0.3%)

**178**	2(0.3%)	2(0.3%)	4(0.3%)
**32**	4(0.5%)	(0%)	4(0.3%)
**18**	2(0.3%)	1(0.2%)	3(0.2%)
**305**	1(0.2%)	2(0.3%)	3(0.2%)
**255**	2(0.3%)	1(0.2%)	3(0.2%)
**91**	1(0.2%)	2(0.3%)	3(0.2%)
**171**	(0%)	3(0.4%)	3(0.2%)
**116**	2(0.3%)	1(0.2%)	3(0.2%)
**254**	(0%)	3(0.4%)	3(0.2%)
**123**	1(0.2%)	2(0.3%)	3(0.2%)
**200**	1(0.2%)	2(0.3%)	3(0.2%)
**242**	1(0.2%)	2(0.3%)	3(0.2%)
**301**	1(0.2%)	1(0.2%)	2(0.2%)
**142**	1(0.2%)	1(0.2%)	2(0.2%)
**506**	1(0.2%)	1(0.2%)	2(0.2%)
**206**	1(0.2%)	1(0.2%)	2(0.2%)
**184**	(0%)	2(0.3%)	2(0.2%)
**233**	1(0.2%)	1(0.2%)	2(0.2%)
**145**	1(0.2%)	1(0.2%)	2(0.2%)
**26**	1(0.2%)	1(0.2%)	2(0.2%)
**23**	1(0.2%)	1(0.2%)	2(0.2%)
**278**	(0%)	2(0.3%)	2(0.2%)
**16**	(0%)	1(0.2%)	1(0.1%)

**469**	1(0.2%)	(0%)	1(0.1%)
**358**	(0%)	1(0.2%)	1(0.1%)
**168**	(0%)	1(0.2%)	1(0.1%)
**504**	(0%)	1(0.2%)	1(0.1%)
**95**	(0%)	1(0.2%)	1(0.1%)
**330**	1(0.2%)	(0%)	1(0.1%)
**253**	1(0.2%)	(0%)	1(0.1%)
**467**	(0%)	1(0.2%)	1(0.1%)
**170**	1(0.2%)	(0%)	1(0.1%)
**471**	1(0.2%)	(0%)	1(0.1%)
**163**	(0%)	1(0.2%)	1(0.1%)
**238**	1(0.2%)	(0%)	1(0.1%)
**143**	1(0.2%)	(0%)	1(0.1%)
**329**	1(0.2%)	(0%)	1(0.1%)
**45**	1(0.2%)	(0%)	1(0.1%)
**355**	1(0.2%)	(0%)	1(0.1%)
**83**	(0%)	1(0.2%)	1(0.1%)
**466**	1(0.2%)	(0%)	1(0.1%)
**512**	1(0.2%)	(0%)	1(0.1%)
**468**	1(0.2%)	(0%)	1(0.1%)
**513**	1(0.2%)	(0%)	1(0.1%)
**470**	1(0.2%)	(0%)	1(0.1%)
**515**	1(0.2%)	(0%)	1(0.1%)

**473**	(0%)	1(0.2%)	1(0.1%)
**218**	1(0.2%)	(0%)	1(0.1%)
**505**	(0%)	1(0.2%)	1(0.1%)
**304**	1(0.2%)	(0%)	1(0.1%)
**47**	(0%)	1(0.2%)	1(0.1%)
**20**	1(0.2%)	(0%)	1(0.1%)
**514**	1(0.2%)	(0%)	1(0.1%)
**280**	1(0.2%)	(0%)	1(0.1%)
**299**	(0%)	1(0.2%)	1(0.1%)
**129**	1(0.2%)	(0%)	1(0.1%)
**Total**	899	926	1825

Table S7. Differences in proportion of resistance to 17 antibiotics in pneumococcal carriage isolates belonging to PCV13 and non-PCV13 serotypes.

Differences in resistance in non-vaccine serotype and vaccine serotypes of pneumococcal isolates % (n)			
Antibiotics	PCV13 serotypes	non-PCV13 serotypes	P-value
Penicillin	75% (615)	18.7% (188)	<0.005
Amoxicillin	3.8% (31)	0	<0.005
Meropenem	11% (90)	0.2% (2)	<0.005
Cefotaxime	6% (49)	0.1% (1)	<0.005
Ceftriaxone	8.7% (71)	0.1% (1)	<0.005
Cefuroxime	14.7% (120)	0.3% (3)	<0.005
Erythromycin	27.9% (228)	5.5% (55)	<0.005
Clindamycin	14.7% (120)	2% (20)	<0.005
Quinupristin-dalfopristin (Synercid)	0	0	1
Linezolid	0	0	1
Co-trimoxazole	83% (680)	57.4% (576)	<0.005
Tetracycline	24.3% (199)	6.1% (61)	<0.005

Doxycycline	24.3% (199)	6.1% (61)	<0.005
Levofloxacin	0	0	1
Chloramphenicol	3.5% (28)	0.3% (3)	<0.005
Rifampin	0	0	1
Vancomycin	0	0	1

There was one isolate with no predicted output for the beta-lactams from adults in the non-PCV13 serotypes. This isolate was taken out of the analysis for calculating the percentages and p-values for beta-lactams. Resistant and intermediate isolates were grouped together into one category for this comparison.

Table S8. Changes in proportion of resistance to 17 antibiotics in pneumococcal carriage isolates stratified by age in pneumococcal isolates belonging to non-vaccine serotypes.

Changes in resistance in non-vaccine serotype pneumococcal isolates between PCV7 and PCV13 era in different age groups % (n)									
Antibiotic	Children ≤2 years of age			Children between 3 to 5 years			Adults over 5 years		
	PCV7 era (N = 197)	PCV13 era (N = 339)	p-value	PCV7 era (N = 70)	PCV13 era (N = 134)	p-value	PCV7 era (N =104)	PCV13 era (N = 161)	p-value
Penicillin	14.3% (28)	24.8% (84)	0.08	4.3% (3)	21.7% (29)	0.02	14.5% (15)	18.2% (29)	1.00
Amoxicillin	0	0	1.00	0	0	1.00	0	0	1.00
Meropenem	0.6% (1)	0.3% (1)	1.00	0	0	1.00	0	0	1.00
Cefotaxime	0	0.3% (1)	1.00	0	0	1.00	0	0	1.00
Ceftriaxone	0	0.3% (1)	1.00	0	0	1.00	0	0	1.00
Cefuroxime	0.6% (1)	0.6% (2)	1.00	0	0	1.00	0	0	1.00
Erythromycin	6.6% (13)	5.1% (17)	1.00	0% (0)	4.5% (6)	0.38	7.7% (8)	6.9% (11)	1.00
Clindamycin	2.1% (4)	1.8% (6)	1.00	0% (0)	0.8% (1)	1.00	2.9% (3)	3.8% (6)	1.00
Quinupristin-dalfopristin (Synercid)	0	0	1.00	0	0	1.00	0	0	1.00
Linezolid	0	0	1.00	0	0	1.00	0	0	1.00

Co-trimoxazole	55.4% (109)	64.7% (219)	0.35	45.8% (32)	62% (83)	0.18	43.3% (45)	54.7% (88)	1.00
Tetracycline	5.6% (11)	6.8% (23)	1.00	0	6.7% (9)	0.18	7.7% (8)	6.2% (10)	1.00
Doxycycline	5.6% (11)	6.8% (23)	1.00	0	6.7% (9)	0.18	7.7% (8)	6.2% (10)	1.00
Levofloxacin	0	0	1.00	0	0	1.00	0	0	1.00
Chloramphenicol	0.6% (1)	0.3% (1)	1.00	0	0.8% (1)	1.00	0	0	1.00
Rifampin	0	0	1.00	0	0	1.00	0	0	1.00
Vancomycin	0	0	1.00	0	0	1.00	0	0	1.00

There was one isolate with no predicted output for the beta-lactams from adults in the PCV13 era. This isolate was taken out of the analysis for calculating the percentages and p-values for beta-lactams. Resistant and intermediate isolates were grouped together into one category for this comparison.

Table S9. Prevalence of ten most prevalent GPSCs from South Africa carriage population in African and Global Context using data from pubMLST and GPS database

**GPSC**	**Predominant Sequence types within GPSC**	**Percentage of isolates from PubMLST data (excluding GPS isolates)**			**Percentage of isolates from GPS data**		
		**South African isolates %(n)**	**African isolates %(n)**	**Non-African countries (n)**	**South African isolates %(n)**	**African %(n)**	**Non-African countries (n)**
**GPSC22**	ST4984	0	100% (1)		50% (174)	99% (346)	Bangladesh (n=2), Cambodia (n=1)
	ST10545	50% (1)	50% (1)				
**GPSC21**	ST347	72.7% (8)	90.9% (10)	Norway (1)	76% (347)	100% (461)	

**GPSC14**	ST6279	100% (41)	100% (41)		82% (399)	84% (411)	Peru (n=40), Thailand (n=9), China (n=6), USA (n=6), Turkey (n=5), Israel (n=5), Lithuania (n=3), Poland (n=2), India (n=2), Brazil (n=1), Russian Federation (n=1), Cambodia (n=1)
**GPSC17**	ST2062	93.6% (146)	94.2% (147)	Australia (1), Belgium (1), Czech Republic (1), UK (6)	87% (453)	95% (491)	Ireland (n=23), New Zealand (n=6), Qatar (n=1), China (n=1)
**GPSC33**	ST4088	100% (1)	100% (1)		78% (150)	100% (194)	
	ST10543	0					
**GPSC5**	ST361	0	37.5% (6)	France (1), Germany (3), Philippines (3), Spain (1), UK (2)	40% (311)	70% (541)	Israel (n=51), USA (n=40), Argentina (n=39), Brazil (n=19), Peru (n=14), Bangladesh (n=14), Pakistan (n=10), Thailand (n=8), China (n=8), Nepal (n=8), Qatar (n=7), Papa New Guinea (n=6), India (n=4), Turkey (n=3), France (n=2), Russian Federation (n=2), Cambodia (n=1), Belarus (n=1), Slovenia (n=1), Canada (n=1)
	ST1447	92.9% (13)	92.9% (13)	Saudi Arabia (1)			
	ST172	13.3% (19)	28.7 (41)	Australia (1), Germany (3), Greece (21), Israel (4), Kenya (7), Malaysia (4), Portugal (1), Spain (1), Thailand (32), UK (7), USA (30)			
**GPSC34**	ST7067	0	100% (1)		45% (104)	99% (233)	India (n=2), Nepal (n=1)
**GPSC37**	ST4929	50% (4)	100% (8)		75% (165)	89% (194)	Argentina (n=7), Bangladesh (n=7), India (n=6), Nepal (n=4), Thailand (n=1), Cambodia (n=1)
	ST2909	75% (6)	100% (8)				

	ST2421	100% (17)	100% (17)				
**GPSC52**	ST5647	100% (3)	100% (3)		91% (118)	100% (131)	
**GPSC13**	ST5073	53.8% (7)	100% (13)		52% (301)	68% (391)	Peru (n=43), Israel (n=31), India (n=20), USA (n=16), Nepal (n=13), Argentina (n=12), Cambodia (n=7), Bangladesh (n=7), China (n=6), Belarus (n=6), Brazil (n=5), Canada (n=4), Poland (n=3), Hungary (n=3), Slovenia (n=3), Trinidad and Tobago (n=3), Turkey (n=3), Malaysia (n=1), Qatar (n=1), Pakistan (n=1), Indonesia (n=1), Lithuania (n=1)
	ST2285	81% (17)	100% (21)				

Table S10. Demographics of the pneumococcal dataset in this study over vaccine periods

Characteristics	Percentage (n/N)		
	PCV7 era	PCV13 era	Total
**n**	899	926	1825
**Gender**			
Female	53.6% (480/896)	53.8% (498/926)	53.7% (978/1822)
Male	46.4% (416/896)	46.2% (428/926)	46.3% (844/1822)
**Age (years)**	PCV7 era	PCV13 era	Total
≤2	57.4% (516/899)	51.7% (479/926)	54.5% (995/1825)
>5	23.5% (211/899)	24.7% (229/926)	24.1% (440/1825)
3--5	19.1% (172/899)	23.5% (218/926)	21.4% (390/1825)
**HIV**	PCV7 era	PCV13 era	
HIV-infected	59.9% (294/491)	68.9%% (427/619)	64.9% (721/1110)
HIV-uninfected	40.1% (197/491)	31.1% (192/619)	35.1% (389/1110)

**Region**			
Agincourt	60.0% (539/899)	60.3% (558/926)	60.1% (1097)
Soweto	40.0% (360/899)	39.7% (368/926)	39.9% (728)
**Year**			
2009	41.6% (374/899)	0	20.5% (374/1825)
2010	40.0% (360/899)	0	19.7% (360/1825)
2011	18.4% (165/899)	20.8% (193/926)	19.6% (358/1825)
2012	0	37.4% (346/926)	19.0% (346/1825)
2013	0	41.8% (387/926)	21.2% (387/1825)

Samples were grouped into two vaccine periods based on the date of sample collection: 1. PCV7 era: included samples collected between June 2009 and July 2011 (after the introduction of PCV7 but before PCV13), 2. PCV13 era: included samples collected between August 2011 and November 2013 (after the introduction of PCV13). The denominator for HIV status varies across the rows because the information on HIV status was available for only a subset of the participants.

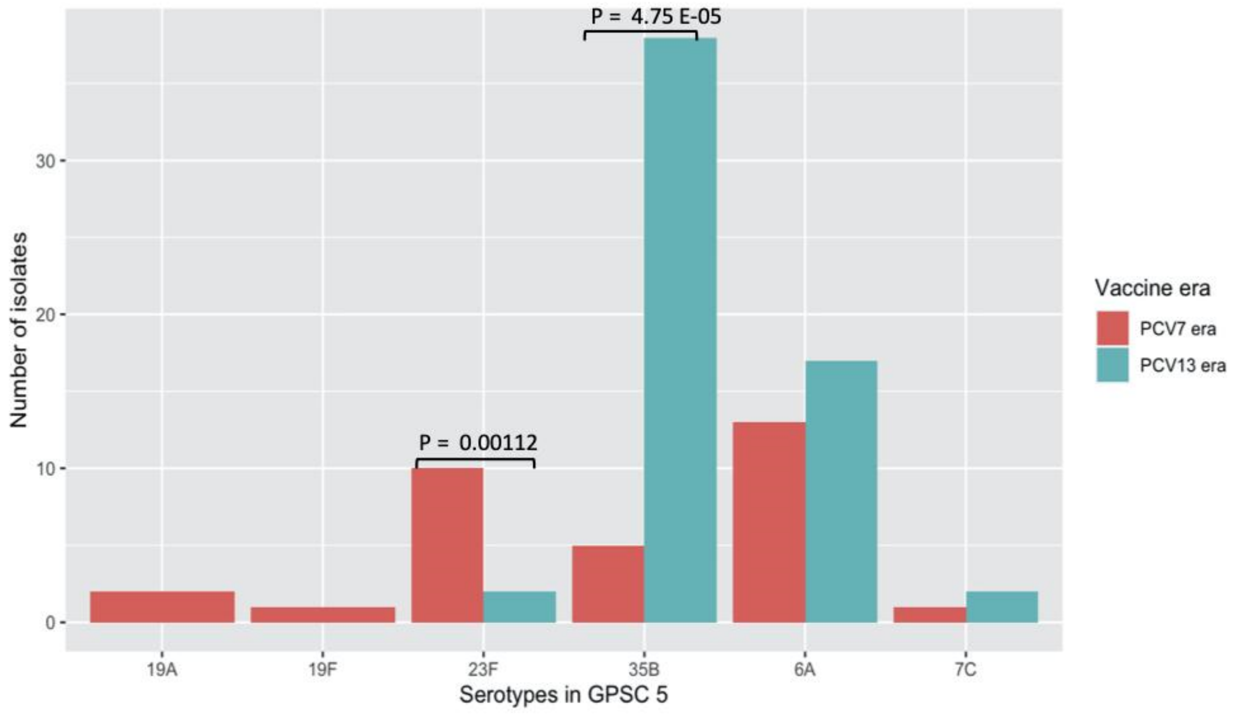

Figure S1. Serotype changes within GPSC 5.

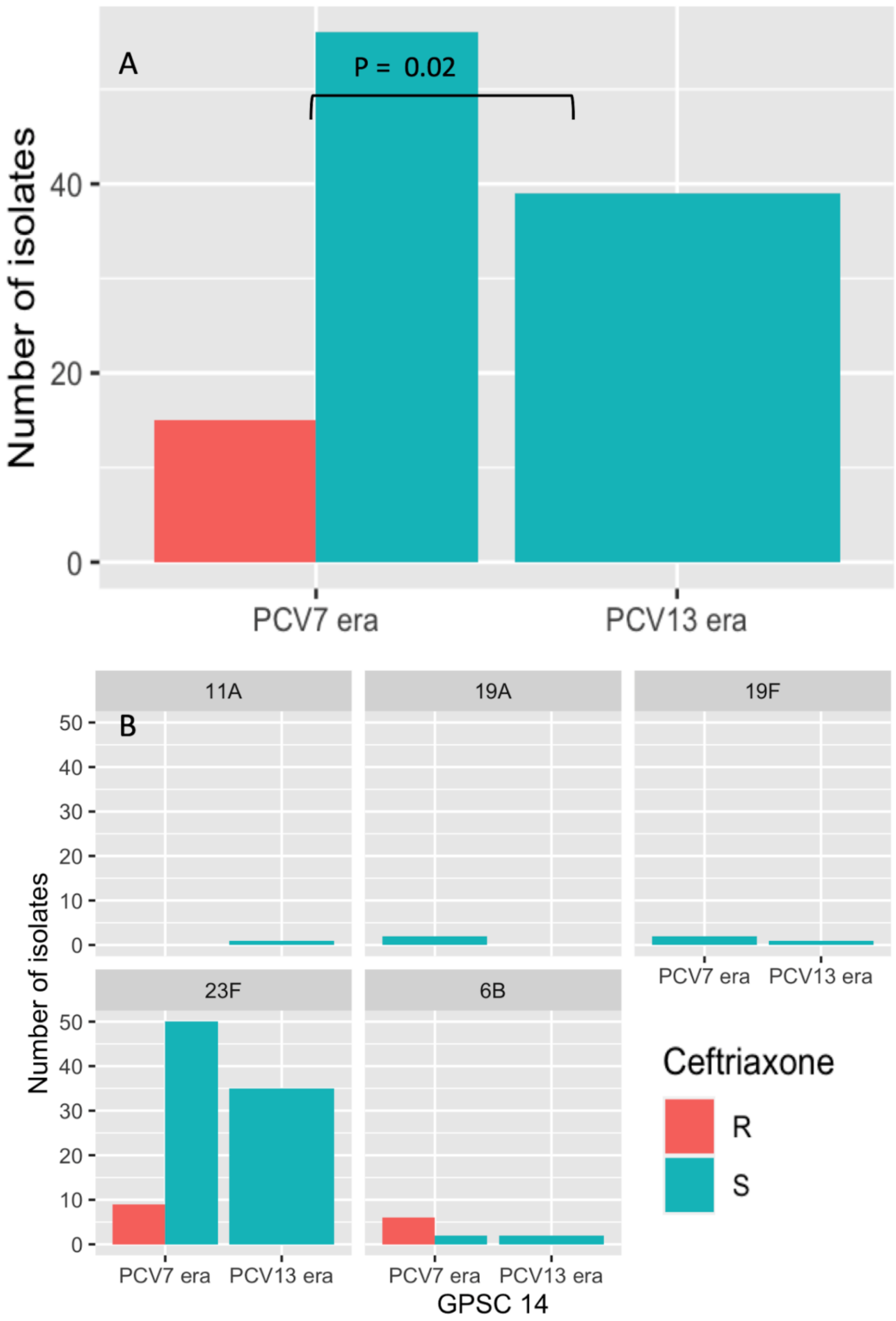

Figure S2. A. Decreasing ceftriaxone resistance within GPSC **14. B.** Serotypes contributing to decreased ceftriaxone resistance in GPSC14

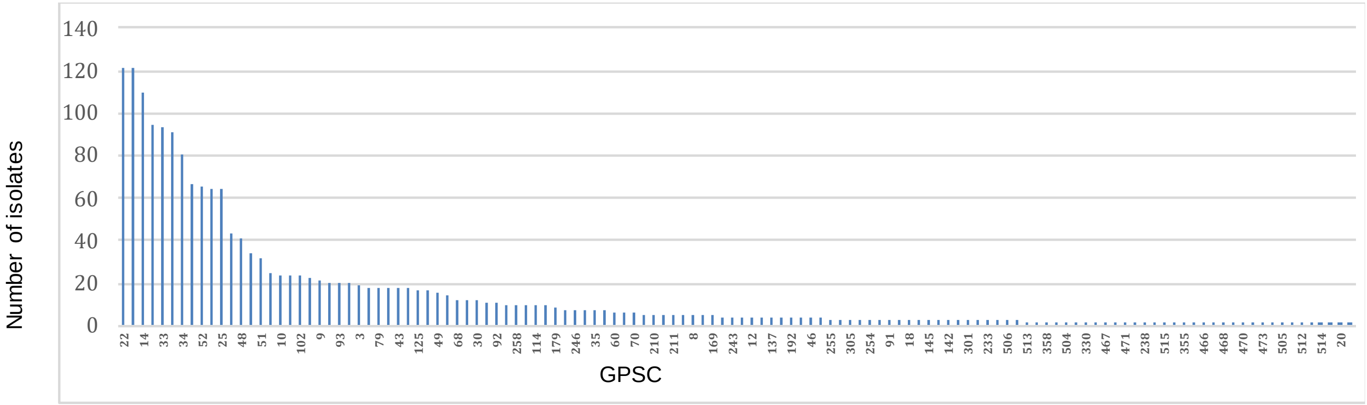

Figure S3. Barchart showing number of isolates belonging to each Global Pneumococcal Sequencing Cluster (GPSCs)

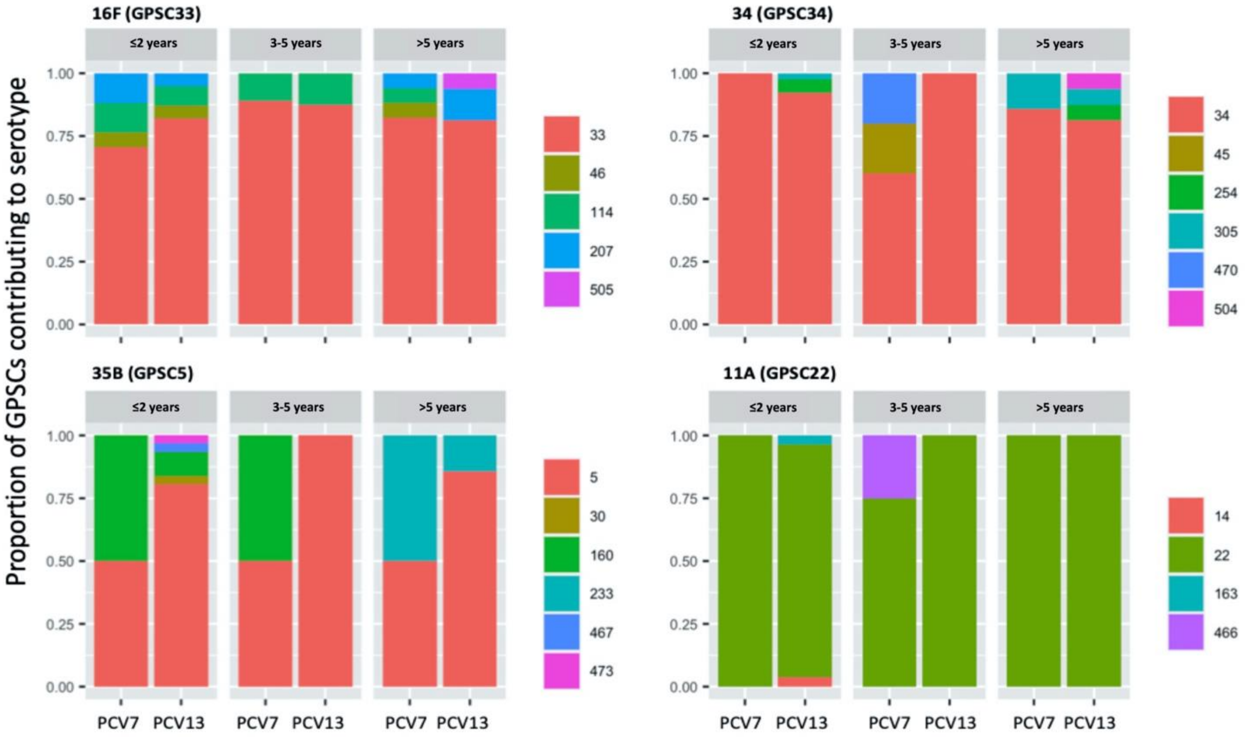

Figure S4. Barchart showing Global Pneumococcal Sequencing Cluster (GPSCs) within serotype 16F, 34, 35B, and 11A
